# Redox Regulation of a Light-Harvesting Antenna Complex in an Anoxygenic Phototroph

**DOI:** 10.1128/mBio.02838-19

**Published:** 2019-11-26

**Authors:** Kathryn R. Fixen, Yasuhiro Oda, Caroline S. Harwood

**Affiliations:** a Department of Plant and Microbial Biology, University of Minnesota, St. Paul, Minnesota, USA; b Department of Microbiology, University of Washington, Seattle, Washington, USA; California Institute of Technology

**Keywords:** *Rhodopseudomonas palustris*, anoxygenic phototroph, light-harvesting antenna, redox

## Abstract

The purple nonsulfur bacterium Rhodopseudomonas palustris is a model for understanding how a phototrophic organism adapts to changes in light intensity because it produces different light-harvesting (LH) complexes under high light (LH2) and low light intensities (LH3 and LH4). Outside of this change in the composition of the photosystem, little is understood about how R. palustris senses and responds to low light intensity. On the basis of the results of transcription analysis of 17 R. palustris strains grown in low light, we found that R. palustris strains downregulate many genes involved in iron transport and homeostasis. The only operon upregulated in the majority of R. palustris exposed to low light intensity was *pucBAd*, which encodes LH4. In previous work, *pucBAd* expression was shown to be modulated in response to light quality by bacteriophytochromes that are part of a low-light signal transduction system. Here we found that this signal transduction system also includes a redox-sensitive protein, LhfE, and that its redox sensitivity is required for LH4 synthesis in response to low light. Our results suggest that R. palustris upregulates its LH4 system when the cellular redox state is relatively oxidized. Consistent with this, we found that LH4 synthesis was upregulated under high light intensity when R. palustris was grown semiaerobically or under nitrogen-fixing conditions. Thus, changes in the LH4 system in R. palustris are not dependent on light intensity *per se* but rather on cellular redox changes that occur as a consequence of changes in light intensity.

## Abstract (precis)

An essential aspect of the physiology of phototrophic bacteria is their ability to adjust the amount and composition of their light-harvesting apparatus in response to changing environmental conditions. The phototrophic purple bacterium R. palustris adapts its photosystem to a range of light intensities by altering the amount and composition of its peripheral LH complexes. Here we found that R. palustris regulates its LH4 complex in response to the cellular redox state rather than in response to light intensity *per se*. Relatively oxidizing conditions, including low light, semiaerobic growth, and growth under nitrogen-fixing conditions, all stimulated a signal transduction system to activate LH4 expression. By understanding how LH composition is regulated in R. palustris, we will gain insight into how and why a photosynthetic organism senses and adapts its photosystem to multiple environmental cues.

## INTRODUCTION

**FIG 1:**
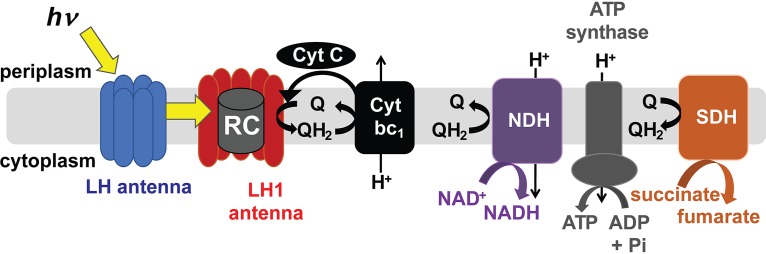
Diagram of cyclic photophosphorylation and electron transfer chain in R. palustris. LH, light harvesting; RC, reaction center; Cyt, cytochrome; NDH, NADH dehydrogenase; SDH, succinate dehydrogenase; Q, quinone; QH_2_, reduced quinone.

The abilities to sense and respond to fluctuating light conditions are an essential aspect of phototroph physiology that is becoming increasingly important as phototrophic organisms are developed as biocatalysts to convert light energy into bioproducts and biofuels. The metabolically versatile purple nonsulfur bacteria (PNSB) have served as model systems for understanding photosynthesis because they carry out anoxygenic photosynthesis using cyclic photophosphorylation, a simple form of photosynthesis. In this process, light energy conversion happens in the reaction center (RC) where electrons are energized by light and cycled through a proton-pumping electron transport chain to maintain a proton gradient used to generate NADH through reverse electron transfer and ATP by ATP synthase (1) (Fig. 1). Most PNSB also have peripheral light-harvesting (LH) complexes, which are involved in capturing light and transferring light energy to the core complex containing the RC. Although peripheral LH complexes are not a requirement for cyclic photophosphorylation, they allow for more efficient light capture under different light intensities (2–4).

The PNSB Rhodopseudomonas palustris is a particularly interesting system since it changes both the amount and composition of its photosystem. Like most PNSB, R. palustris makes more carotenoids (Car) and bacteriochlorophyll *a* (Bchl*a*), the light-capturing pigments used in its photosystem, in response to a decrease in light intensity (5, 6). R. palustris also encodes multiple peripheral light-harvesting complexes, and it adjusts the composition of its LH complexes in response to changes in light intensity (7–12). In R. palustris subjected to high light (30 μmol photons/m^2^/s), the predominant peripheral LH complex is the LH2 complex, a ring of α/β peptide pairs that each bind one Car, one Bchl*a* that absorbs light at ∼800 nm, and two Bchl*a* that absorb light at ∼850 nm. Under low light intensity (4 μmol photons/m^2^/s), R. palustris synthesizes an alternative LH2-like complex known as LH4, which absorbs only at ∼800 nm (13). LH4 is thought to make light harvesting more efficient under low light intensities, and although it is not required for growth under light intensities as low as 4 μmol photons/m^2^/s, it is required for growth under extremely low light intensities (<1 μmol photons/m^2^/s) (3, 6, 14). At least one strain of R. palustris, BisA53, has also been shown to make an LH3 complex when exposed to low light intensity, which absorbs 800- and 820-nm light (15, 16).

**FIG 2:**
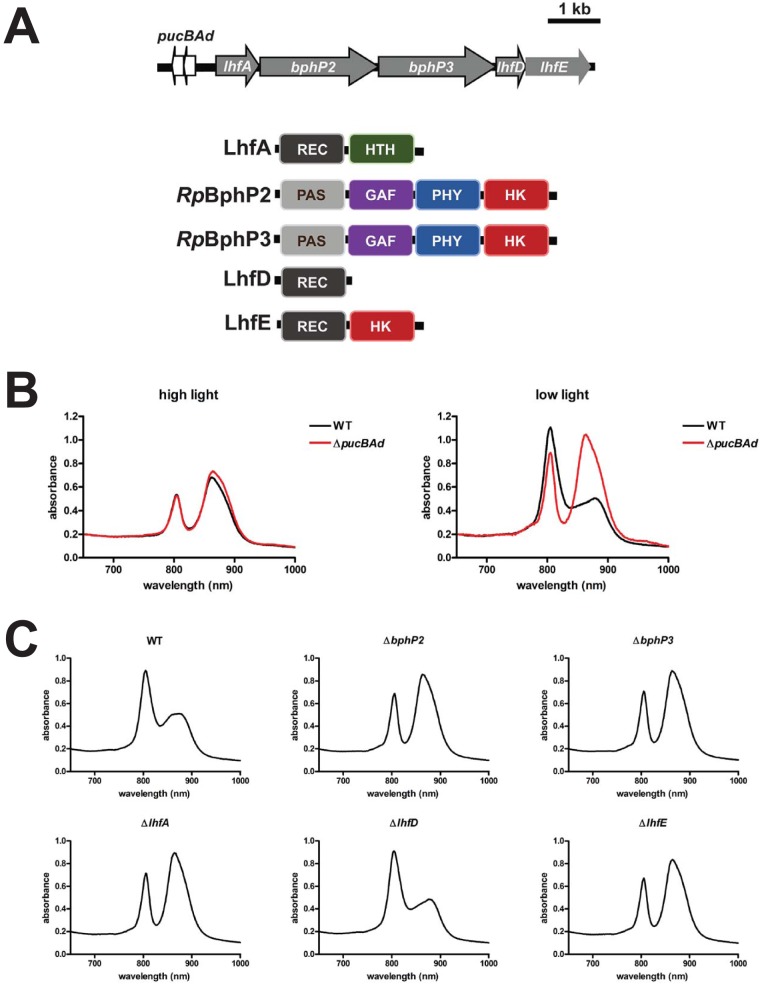
*Rp*BphP2, *Rp*BphP3, LhfA, and LhfE are required for LH4 expression. (A) Genes adjacent to *pucBAd*, which encodes the alpha and beta peptide of LH4, and their predicted domain structure. The domains are indicated as follows: REC, receiver domain; HTH, helix-turn-helix domain; PAS, Per Arnt Sim domain; GAF, cGMP phosphodiesterases, adenylyl cyclases, and FhlA domain; PHY, phytochrome domain; HK, histidine kinase domain. (B) Representative absorption spectra of intact WT R. palustris CGA009 and R. palustris Δ*pucBAd* grown anaerobically and exposed to either high light intensity (30 μmol photons/m^2^/s) or low light intensity (4 μmol photons/m^2^/s). (C) Representative absorption spectra of intact WT R. palustris and R. palustris with a deletion of either *lhfA* (*rpa3014*), *bphP2* (*rpa3015*), *bphP3* (*rpa3016*), *lhfD* (*rpa3017*), or *lhfE* (*rpa3018*) grown anaerobically and exposed to low light intensity (4 μmol photons/m^2^/s).

The two peptides that make up the LH4 complex are encoded by the operon *pucBAd* (*rpa3013* and *rpa3012*). Adjacent to this operon is a set of genes that encodes a putative signal transduction system (Fig. 2A). Two of these genes, *bphP2* (*rpa3015*) and *bphP3* (*rpa3016*), are required for LH4 synthesis in cells exposed to low light (9–12). These genes encode photosensory regulatory proteins known as bacteriophytochromes *Rp*BphP2 (R. palustris BphP2) and *Rp*BphP3 that sense changes in light quality (10). Under semiaerobic conditions, the ability of *Rp*BphP2/P3 to sense light quality plays a role in inducing LH4 synthesis in response to red light (10, 11). However, the ability to sense light quality is dispensable for sensing low light intensity under anaerobic conditions, and it is unclear how R. palustris senses changes in light intensity to regulate LH4 synthesis under these conditions (11).

To gain insight into how R. palustris senses and responds to low light, changes in gene expression for 17 R. palustris strains grown under high light (30 μmol photons/m^2^/s) and low light (4 μmol photons/m^2^/s) were analyzed. From this analysis, we found that *pucBAd* was the only operon upregulated in low light in at least 14 of the 17 strains tested. By further characterizing the signal transduction system controlling expression of *pucBAd*, we found that one of its components is redox sensitive and that redox sensitivity was required for LH4 synthesis in cells exposed to low light. We also found that LH4 synthesis was induced under high light when cells were grown semiaerobically or under conditions that require nitrogen fixation, which are conditions expected to alter the redox state of the cell to make it more oxidizing. From this, we conclude that LH4 synthesis is controlled by redox rather than light intensity *per se*.

## RESULTS

### Effects of exposure to low light intensity on gene expression in R. palustris.

Even though changes in the amount and composition of the photosystem in R. palustris cells exposed to different light intensities have been well documented, very little is understood about how light intensity affects other aspects of R. palustris physiology. To gain insight into how light intensity may affect gene expression, we used a large data set from reference 8 comprised of RNA-seq data from 17 closely related strains of R. palustris grown under high light (30 μmol photons/m^2^/s) and low light (4 μmol photons/m^2^/s). Genes from different strains were put into orthologous groups using OrthoMCL (17). The expression level of genes in each orthologous group was determined for each strain, and orthologous genes whose expression levels were significantly different under high light versus low light intensity were determined (see Table S1 in the supplemental material).

10.1128/mBio.02838-19.2 TABLE S1 Change in gene expression for orthologous genes in 17 R. palustris strains exposed to low light and high light intensity. Download Table S1, XLSX file, 1.1 MB. Copyright © 2019 Fixen et al. 2019 Fixen et al. This content is distributed under the terms of the Creative Commons Attribution 4.0 International license.

Of the orthologous genes that were differentially expressed, 114 orthologous genes were downregulated more than twofold in at least 14 of the 17 strains grown at low light. As shown in Table S2, half of the genes (56%) are related to iron transport and metabolism or are in an operon with genes related to iron transport and metabolism. Other orthologous genes implicated in metal ion transport (*rpa0220* and *rpa2333-2338*) and FeS cluster biosynthesis (*rpa1606* and *rpa2468-2470*) were also downregulated in strains exposed to low light, suggesting that maintaining metal homeostasis is important under low light intensity.

10.1128/mBio.02838-19.3 TABLE S2 Genes downregulated under low light intensity in at least 14 of 17 R. palustris strains. Download Table S2, XLSX file, 0.04 MB. Copyright © 2019 Fixen et al. 2019 Fixen et al. This content is distributed under the terms of the Creative Commons Attribution 4.0 International license.

Surprisingly, only seven orthologous genes were upregulated more than twofold in at least 14 of the 17 strains grown with low light (Table S3). This includes the operon encoding the LH4 complex, *pucBAd* (*rpa3013* and *rpa3012*), as well as orthologs encoding the LuxR-family transcription regulator, *rpa3014*, and a hypothetical protein, *rpa3011*, which bookend *pucBAd* on the chromosome. The only strains that did not upregulate these orthologs were DSM8283, which does not encode these genes, and BIS3 and DCP3, as reported previously (8). In addition, orthologs of *pucBc* (*rpa3009*), which encodes the β peptide of a putative LH3 complex were also upregulated. Four strains, CGA009, 0001L, ATCC 17007, and CEA001, do not encode an intact α peptide of the putative LH3 complex, so these orthologs were not included in our analysis. These data are consistent with LH4 and LH3 complexes being found predominantly in R. palustris exposed to low light intensities. In addition, orthologs of two genes encoding hypothetical proteins, *rpa3035* and *rpa3587*, were upregulated in at least 14 of the 17 strains.

10.1128/mBio.02838-19.4 TABLE S3 Genes upregulated under low light intensity in at least 14 of 17 R. palustris strains. Download Table S3, XLSX file, 0.01 MB. Copyright © 2019 Fixen et al. 2019 Fixen et al. This content is distributed under the terms of the Creative Commons Attribution 4.0 International license.

To confirm that the increase in *pucBAd* expression in low light results in LH4 production, we assayed LH4 synthesis in cells grown in high light or low light. LH4 synthesis can be assayed by measuring the ratio of whole-cell absorbance at 800 nm to absorbance at 860 nm. When this ratio is greater than 1, LH4 predominates in the membrane, and when this ratio is less than 1, LH2 predominates (9, 13). As shown in Fig. 2B and Table 1, R. palustris grown in low light had an increase in absorption of 800-nm light and a decrease in absorption of 860-nm light, which is consistent with an increase in LH4 complexes in the membrane. An in-frame deletion of *pucBAd* confirmed that this change in the absorption spectra under low light intensity was due to expression of *pucBAd* (Fig. 2B and Table 1). Taken together, these data indicate that *pucBAd* expression is an easily assayed marker of exposure to low light intensity.

**TABLE 1:** The 800-nm/860-nm absorption ratio of wild-type and mutant strains of R. palustris exposed to low light intensity (4 μmol photons/m^2^/s)^*a*^

Genotype	800-nm/860-nm absorption ratio (SD)^*b*^
WT	2.0 (0.2)
Δ*pucBAd*	0.7 (0.01)
Δ*lhfA*	0.7 (0.02)
Δ*lhfA* pBBRMCS-5	0.7 (0.01)
Δ*lhfA* p-*lhfA*	1.7 (0.05)
Δ*bphP2*	0.7 (0.02)
Δ*bphP3*	0.7 (0.02)
Δ*lhfD*	1.9 (0.01)
Δ*lhfE*	0.7 (0.01)
Δ*lhfE* pBBPgdh	0.7 (0.04)
Δ*lhfE* p-*lhfE*	1.7 (0.01)

a All strains were grown in non-nitrogen-fixing minimal medium with 20 mM acetate. Light was provided from a 15-W incandescent bulb.

b All values are the averages from three or more independent experiments. SD, standard deviation.

### A putative phosphorelay controls expression of *pucBAd*, the operon encoding LH4.

To understand how R. palustris senses low light, we decided to focus on regulation of *pucBAd* expression by a putative signal transduction system that is encoded next to *pucBAd* on the chromosome (18) (Fig. 2A). We also decided in view of evidence presented below to name this the Lhf system for light-harvesting four. Two of the Lhf system genes, *bphP2* and *bphP3*, have been shown to be required for LH4 synthesis under low light intensity (9, 10, 12). To determine whether the other genes in this operon are required for LH4 synthesis in low light conditions, we made in-frame deletions in *lhfA* (*rpa3014*), *lhfD* (*rpa3017*), and *lhfE* (*rpa3018*) and assayed LH4 synthesis. As shown in Fig. 2C and Table 1, only deletions in *lhfA* or *lhfE* disrupted LH4 synthesis in cells grown under low light, and LH4 synthesis was restored in these mutants when a wild-type (WT) allele was provided in *trans*. Although *lhfD* was not required to sense light intensity, it has been shown to interact with *Rp*BphP2/P3, and it serves as the cognate response regulator for these two proteins in phosphotransfer (10, 19).

LH4 synthesis occurs in response to red light, and under this condition, *Rp*BphP2/P3 are largely in a dephosphorylated, activating, state (10). To mimic this state and potentially create a strain with constitutive expression of *pucBAd*, R. palustris strains expressing a variant of either *Rp*BphP2 or *Rp*BphP3 with an alanine substitution in the histidine kinase domain at the conserved His532 or His547, respectively, were constructed. As shown in Table 2, increased LH4 synthesis occurred in these strains grown in high light, consistent with *in vitro* data suggesting that dephosphorylated *Rp*BphP2/P3 are active. However, a much greater amount of LH4 was synthesized when these strains were grown in low light, suggesting that there is a low light signal, not dependent on unphosphorylated *Rp*BphP2/P3, that controls LH4 expression. In addition, deletion of *lhfE* in a strain expressing *Rp*BphP2^H532A^ disrupted LH4 synthesis in cells exposed to low light, suggesting that LhfE acts downstream of *Rp*BphP2/P3 (Table 2). LhfE contains a receiver (REC) domain and a histidine kinase domain (Fig. 2A). R. palustris strains expressing a variant of LhfE with an alanine substitution at the conserved His185 in its histidine kinase domain or a variant of LhfA with an alanine substitution at the conserved Asp70 in its receiver domain were unable to synthesize LH4 when exposed to low light intensity (see Fig. S1 in the supplemental material).

**TABLE 2:** The 800-nm/860-nm absorption ratio of wild-type and mutant strains of R. palustris exposed to different light intensities^*a*^

Genotype	800-nm/860-nm absorption ratio (SD)^*b*^	
	High light intensity^*c*^	Low light intensity^*d*^
WT	0.7 (0.03)	2.1 (0.2)
Δ*bphP2*	0.7 (0.02)	0.7 (0.02)
*bphP2*^H532A^	1.0 (0.02)	2.2 (0.4)
Δ*bphP3*	0.7 (0.01)	0.7 (0.03)
*bphP3*^H547A^	1.0 (0.15)	2.2 (0.2)
*bphP2*^H532A^ *bphP3*^H547A^	1.0 (0.2)	2.3 (0.4)
*bphP2*^H532A^ Δ*lhfE*	0.7 (0.04)	0.7 (0.01)

a All strains were grown in non-nitrogen-fixing minimal medium with 20 mM acetate.

b All values are the averages from three independent experiments. SD, standard deviation.

c High light intensity (30 μmol photons/m^2^/s) was generated from a 60-W incandescent bulb.

d Low light intensity (4 μmol photons/m^2^/s) was generated from a 15-W incandescent bulb.

10.1128/mBio.02838-19.1 FIG S1 Representative absorption spectra of intact WT R. palustris, R. palustris with a deletion of either *lhfA* (*rpa3014*) or *lhfE* (*rpa3018*), and R. palustris carrying *lhfA*^D70A^ or *lhfE*^H185A^ grown anaerobically in non-nitrogen-fixing medium and exposed to low light intensity (4 μmol photons/m^2^/s). Download FIG S1, TIF file, 1.1 MB. Copyright © 2019 Fixen et al. 2019 Fixen et al. This content is distributed under the terms of the Creative Commons Attribution 4.0 International license.

*lhfA* encodes a putative transcription regulator and is likely the last step in the signal transduction cascade regulating transcription of *pucBAd*. Wild-type R. palustris and an *lhfA* mutant containing a transcription reporter with *lacZ* under the control of the *pucBAd* promoter were constructed, and β-galactosidase activity in these strains was determined in cells grown in low light. As shown in Table 3, an increase in β-galactosidase activity was observed with this reporter compared to the vector control, and this activity was dependent on *lhfA*, since β-galactosidase activity dropped to levels similar to that of the vector control in the *lhfA* deletion mutant. This indicates that *lhfA* is required to activate *pucBAd* expression in cells exposed to low light intensity.

**TABLE 3:** Gene expression of *pucBAd* requires LhfA under low light intensity^*a*^

Genotype	β-Galactosidase activity (nmol/min/mg total protein)^*b*^
WT pHRP309	264 ± 16
WT pHRP309-P*_pucBAd_*::*lacZ*	1,440 ± 24
Δ*lhfA* pHRP309	347 ± 6
Δ*lhfA* pHRP309-P*_pucBAd_*::*lacZ*	341 ± 19

a All strains were grown in non-nitrogen-fixing medium under low light intensity (4 μmol photons/m^2^/s) provided from a 15-W incandescent bulb.

b Values are the averages ± standard deviations for three replicates.

### Redox sensitivity of LhfE is required for LH4 synthesis under low light intensity.

*Rp*BphP2, *Rp*BphP3, LhfE, and LhfA are part of signal transduction system that activates expression of *pucBAd* in response to signals from light. Although *Rp*BphP2/P3 sense specific wavelengths of light, we have shown that this ability is not required for LH4 synthesis under low-intensity, white light (11). This indicates that R. palustris is not sensing light intensity as a change in light quality via *Rp*BphP2/P3. This leaves LhfE as an unexplored player in the Lhf signal transduction system.

**FIG 3:**
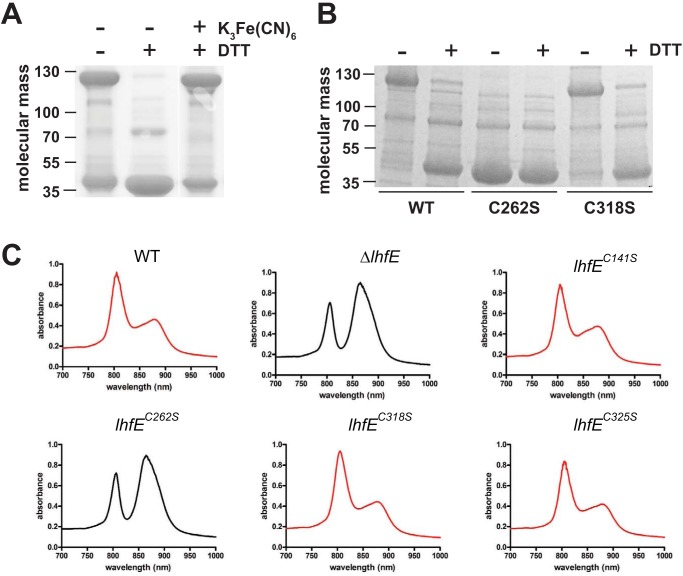
LhfE is redox sensitive, and redox sensitivity is required for regulation of LH4 synthesis. (A) Protein stain of nonreducing SDS-PAGE of purified His_6x_-LhfE treated with (+) or without (−) dithiothreitol (DTT) or potassium ferric cyanide [K_3_Fe(CN)_6_]. Molecular mass is shown in kilodaltons. (B) Protein stain of nonreducing SDS-PAGE of purified His_6x_-LhfE and variants that contained a serine substitution at either Cys262 or Cys318. Purified protein was incubated with (+) or without (−) DTT. Molecular mass is shown in kilodaltons. (C) Representative absorption spectra of intact WT R. palustris, R. palustris with a deletion of *lhfE* or R. palustris encoding variants of LhfE grown anaerobically and exposed to low light intensity (4 μmol photons/m^2^/s).

In other PNSB, light intensity is sensed as a redox signal (20, 21). In the PNSB Rhodobacter sphaeroides and Rhodobacter capsulatus, light-sensing regulatory proteins can sense signals from light and redox using a variety of mechanisms, including binding cofactors like heme, directly interacting with quinones in the membrane, or using a thiol-based redox switch (21–27). Based on its amino acid sequence, LhfE is not predicted to bind heme, flavins, or quinones. However, it does have four cysteines (Cys141, Cys262, Cys318, and Cys325), and it seemed possible that LhfE could be functioning as a thiol-based redox sensor. To test this, we purified His-tagged LhfE under aerobic conditions, subjected it either to ultrafiltration or exposure to air overnight, and ran it on a nonreducing SDS-polyacrylamide gel as described previously (28). The expected molecular mass of LhfE is 42 kDa. As shown in Fig. 3, although some monomeric LhfE is present, the majority of the protein runs at around 130 kDa. Incubation of the sample with the reducing agent dithiothreitol (DTT) resulted in the majority of the protein running at the predicted molecular mass of 42 kDa (Fig. 3A). Formation of the higher-molecular-weight form of LhfE was restored by the further addition of the oxidizing agent potassium ferricyanide [K_3_Fe(CN)_6_] (Fig. 3A). This indicates that LhfE is redox sensitive and suggests that the redox environment can change the oligomerization state of LhfE in a manner that is reversible.

Of the four Cys found in LhfE, only Cys262 and Cys318 are conserved in LhfEs from all R. palustris strains that have them. To see whether Cys262 and Cys318 are involved in mediating the redox sensitivity of LhfE, WT LhfE and variants of LhfE with either a C262S or C318S substitution were purified as described above and run on a nonreducing SDS-polyacrylamide gel. As shown in Fig. 3B, LhfE^C318S^ formed a band of about 130 kDa under nonreducing conditions, but LhfE^C262S^ did not. This indicates that Cys262 is required for the redox responsiveness of LhfE *in vitro.* To determine whether Cys262 is also required for LhfE activity *in vivo* and to rule out a possible role for the other Cys residues, R. palustris strains were constructed that expressed variants of LhfE with serine substitutions at Cys141, Cys262, Cys318, or Cys325. As shown in Fig. 3C, only the strain expressing LhfE^C262S^ was unable to synthesize LH4 when grown under low light intensity, indicating that Cys262 is required for LhfE activity *in vivo* and that the redox sensitivity of LhfE is required for LH4 synthesis (Fig. 3).

### R. palustris alters its LH composition in response to other environmental conditions besides light intensity.

Our data suggest that LH4 expression is controlled by a redox signal generated by exposure to low light. Light intensity is expected to affect the redox state of the quinone pool and thus the generation of NADH through reverse electron transfer (29). We determined the ratio of [NAD^+^] to [NADH] in R. palustris cells grown in high light or low light. As shown in Table 4, the [NAD^+^]/[NADH] ratio is higher when cells are exposed to low light intensity than high light intensity, indicating that the NAD^+^/NADH pool becomes more oxidized under low light intensity. This suggests that signals from the electron transport chain, either the redox state of the quinone pool or the amount of reducing equivalents available, probably regulate LH4 synthesis.

**TABLE 4:** Effects of carbon source, growth conditions, and light intensity on the [NAD^+^]/[NADH] ratio in wild-type R. palustris

Carbon source^*a*^	Growth conditions	Light intensity (μmol/m^2^/s)^*b*^	[NAD^+^]/[NADH] ratio (SD)^*c*^
Acetate	Non-nitrogen-fixing, anaerobic	30	5.1 (2.0)
	Non-nitrogen-fixing, anaerobic	4	18.2 (3.2)
	Non-nitrogen-fixing, semiaerobic	30	17.7 (3.6)
	Nitrogen-fixing, anaerobic	30	8.5 (3.8)
	Nitrogen-fixing, anaerobic	4	21.3 (3.3)

Succinate	Nitrogen-fixing, anaerobic	30	5.4 (0.3)

a Either 20 mM acetate or 10 mM succinate was added as a carbon source.

b High light intensity (30 μmol photons/m^2^/s) was generated from a 60-W incandescent bulb, and low light intensity (4 μmol photons/m^2^/s) was generated from a 15-W incandescent bulb.

c All values are the averages from three independent experiments. SD, standard deviation.

**FIG 4:**
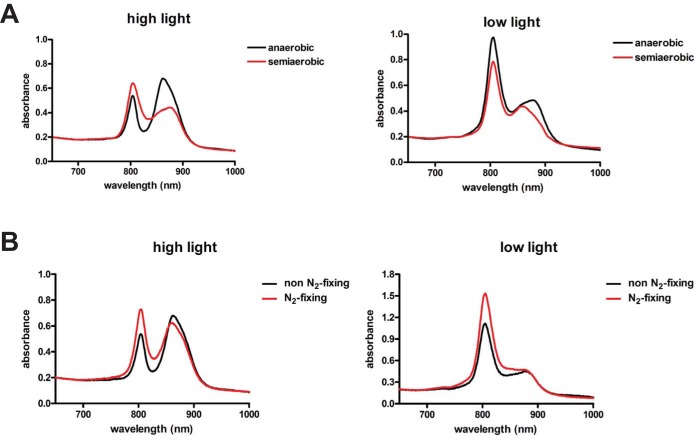
LH4 synthesis is upregulated under semiaerobic and nitrogen-fixing conditions. (A) Absorption spectra of intact WT R. palustris grown anaerobically or semiaerobically and exposed to either high light intensity (30 μmol photons/m^2^/s) or low light intensity (4 μmol photons/m^2^/s). (B) Absorption spectra of intact WT R. palustris grown anaerobically under non-nitrogen-fixing or nitrogen-fixing conditions and exposed to either high light intensity (30 μmol photons/m^2^/s) or low light intensity (4 μmol photons/m^2^/s).

Nitrogen fixation and respiration are processes known to consume reducing power, and we reasoned that R. palustris should therefore synthesize LH4 under high light conditions when grown semiaerobically or under nitrogen-fixing conditions. As shown in Table 4, the [NAD^+^]/[NADH] ratio was higher in cells grown semiaerobically compared to cells grown anaerobically in the presence of high light intensities, indicating that the NAD^+^/NADH pool is more oxidized under semiaerobic conditions. Consistent with this, LH4 synthesis was observed in wild-type R. palustris cells incubated in high light when cells were grown semiaerobically (Fig. 4). Nitrogen-fixing conditions resulted in only a slightly more oxidized NAD^+^/NADH pool than non-nitrogen-fixing conditions when cells were incubated in high light (Table 4), but an increase in LH4 synthesis was still observed under nitrogen-fixing conditions (Fig. 4). Intact *lhfE* and *lhfA* genes were required for LH4 synthesis in semiaerobic and nitrogen-fixing conditions (data not shown). Additionally, semiaerobic or nitrogen-fixing conditions had an additive effect with low light intensity in stimulating LH4 synthesis (Fig. 4).

Reverse electron transfer is particularly important for growth on a substrate like succinate. Succinate is oxidized to fumarate by succinate dehydrogenase, which passes electrons to ubiquinone, and NADH must be generated by reverse electron transfer to prevent overreduction of the electron transfer chain under photosynthetic conditions. Therefore, growth on succinate should lead to a more reduced quinone pool and more NADH generated by reverse electron transfer. If LH4 synthesis is regulated by the redox state of the electron transfer chain, less LH4 should be synthesized when succinate is used as a growth substrate. As shown in Table 5, when R. palustris is grown under conditions where LH4 synthesis is upregulated (i.e., nitrogen-fixing, high light or non-nitrogen-fixing, low light) but succinate is used as growth substrate instead of acetate, LH4 synthesis is somewhat reduced as measured by the ratio of absorbance of 800-nm/860-nm light. Consistent with this, the [NAD^+^]/[NADH] ratio in cells grown under nitrogen-fixing conditions with succinate as a growth substrate is similar to the [NAD^+^]/[NADH] ratio in cells grown under non-nitrogen-fixing conditions in the presence of high light (Table 4). These data indicate that LH4 synthesis is likely regulated by the redox state of the electron transport chain and is not restricted to conditions in which light is limiting.

**TABLE 5:** Effect of carbon source on 800-nm/860-nm absorption ratio of R. palustris exposed to different light intensities

Genotype	Carbon source^*a*^	Growth medium	800-nm/860-nm absorption ratio (SD)^*b*^	
			High light intensity^*c*^	Low light intensity^*d*^
WT	Acetate	Non-nitrogen-fixing	0.8 (0.003)	2.5 (0.2)^*e*^
	Succinate	Non-nitrogen-fixing	0.8 (0.003)	2.1 (0.3)^*e*^
	Acetate	Nitrogen-fixing	1.2 (0.1)^*f*^	3.1 (0.2)^*g*^
	Succinate	Nitrogen-fixing	0.8 (0.003)^*f*^	2.5 (0.01)^*g*^

Δ*pucBAd*	Acetate	Non-nitrogen-fixing	0.7 (0.01)	0.8 (0.01)
	Succinate	Non-nitrogen-fixing	0.7 (0.1)	0.8 (0.05)
	Acetate	Nitrogen-fixing	0.8 (0.01)	0.8 (0.08)
	Succinate	Nitrogen-fixing	0.8 (0.1)	0.8 (0.02)

a Either 20 mM acetate or 10 mM succinate was added as a carbon source.

b SD, standard deviation.

c High light intensity (30 μmol photons/m^2^/s) was generated from a 60-W incandescent bulb.

d Low light intensity (4 μmol photons/m^2^/s) was generated from a 15-W incandescent bulb.

e *P* = 0.06 between WT acetate and WT succinate at low light (non-nitrogen-fixing).

f *P* = 0.03 between WT acetate and WT succinate at high light (nitrogen-fixing).

g *P* = 0.003 between WT acetate and WT succinate at low light (nitrogen-fixing).

## DISCUSSION

In trying to understand how R. palustris senses low light, we characterized a putative signal transduction system that is responsible for regulating expression of *pucBAd.* Two bacteriophytochromes, *Rp*BphP2 and *Rp*BphP3, were known to be required for LH4 synthesis, but the other members of this signaling system were unknown. It was also unclear how this system was sensing low light, since the ability of *Rp*BphP2/P3 to sense light quality (red light) is not required to regulate LH4 synthesis in response to low light (11), even though the proteins themselves need to be present. We found that two proteins encoded by genes next to *bphP2* and *bphP3*, LhfE (Rpa3018) and LhfA (Rpa3014), are also required for expression of *pucBAd*. We found that LhfA, a putative transcription regulator, is either directly or indirectly controlling transcription of *pucBAd*. We also found that LhfE is redox sensitive and that this redox sensitivity is required for LH4 synthesis in cells exposed to low light. This redox response is mediated by a conserved cysteine, Cys262, in LhfE. However, it is still unclear whether Cys262 mediates formation of a disulfide bond or is modified through another mechanism (e.g., glutathionylation) and how oxidation of Cys262 affects the oligomerization state of LhfE *in vivo*. Future work will focus on understanding how BphP2/P3 interact with LhfE and the role of Cys262 in mediating redox sensing of LhfE.

**FIG 5:**
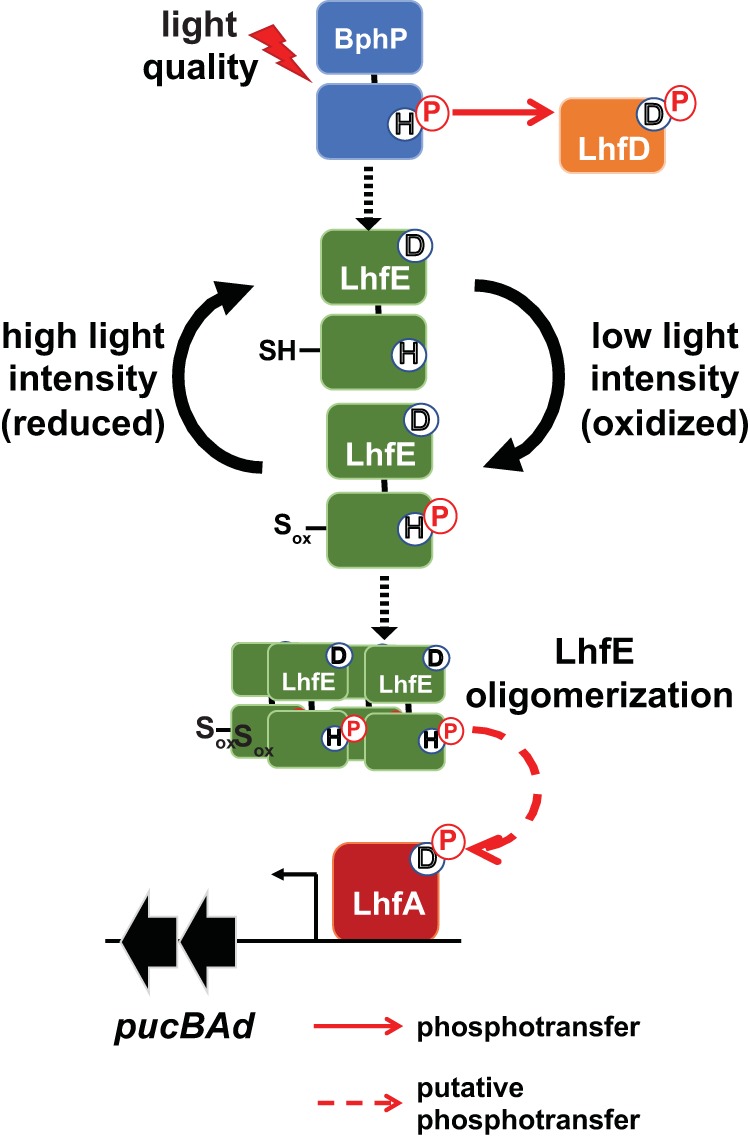
Model for signal transduction cascade leading to the LH4 low light response under anaerobic conditions. Bacteriophytochromes *Rp*BphP2 and *Rp*BphP3 (BphP) sense light quality (red light) and phosphorylate LhfD (10). It is unclear how *Rp*BphP2 and *Rp*BphP3 interact with LhfE, and this uncertainty is indicated as a black, dotted arrow. Under low light, semiaerobic or nitrogen-fixing conditions, the cellular environment becomes more oxidized, and this leads to oxidation of Cys262 (S_ox_) in LhfE, which could potentially form a disulfide bond or undergo modification in some other way such as S-glutathionylation. This is turn leads to oligomerization of LhfE; however, as shown by the black, dotted arrow, the oligomerization state of LhfE is still uncertain. This would render LhfE proficient to transphosphorylate and activate the LhfA transcription factor. P, phosphate; H, histidine; D, aspartic acid.

From these findings, we can present a still incomplete model in which LhfE senses a redox signal generated under low light intensity and *Rp*BphP2/P3 senses light quality to activate LhfA and upregulate expression of *pucBAd* (Fig. 5). Our evidence suggests that a redox signal is the main signal responsible for regulating LH4 synthesis. *In vitro* data suggest that *Rp*BphP2/P3 are largely in a light-induced dephosphorylated state when they are active in signaling and that LhfD (*rpa3017*) acts as a sink for phosphate donated by *Rp*BphP2/P3 (10). At this point, we do not know how dephosphorylated *Rp*BphP2/P3 may influence the activity of LhfE, and we have indicated this uncertainty with a dotted black arrow in Fig. 5.

Like other PNSB, R. palustris exhibits increased pigmentation and photosystem production when exposed to low light intensities. In other PNSB like R. sphaeroides and R. capsulatus, these changes are in part reflected at the transcription level, and increased expression of components of the core complex are observed under low light intensities (30, 31). After looking at changes in gene expression of 17 R. palustris strains grown in low light, we were surprised to find that only one intact operon, *pucBAd*, was upregulated in most strains of R. palustris. It is possible that some of the physiological changes observed under low light intensity are not regulated at the level of transcription but are perhaps controlled through posttranscriptional regulation. Posttranscriptional regulation of the tetrapyrrole pathway, of which Bchl biosynthesis is one branch, has been shown in other photosynthetic organisms (32, 33). This type of regulation could lead to greater flux through the tetrapyrrole pathway and more Bchl biosynthesis without a corresponding increase in expression of genes related to Bchl biosynthesis.

An increase in flux through the tetrapyrrole pathway could also explain the enrichment of downregulated genes involved in iron transport and metabolism. Heme synthesis is another branch of the tetrapyrrole pathway. In other *Rhizobiales* related to R. palustris, iron homeostasis is regulated by the iron response regulator, Irr, which senses intracellular iron indirectly by monitoring heme biosynthesis (34–37). It is possible that increased flux through the tetrapyrrole pathway leads to an increase in heme production under low light intensity, which in turn would lead to decreased expression of genes involved in iron transport and metabolism. Further work is needed to understand if there is greater flux through this pathway and how this increased flux is achieved.

Decreased light intensity leads to a more oxidized quinone pool, and we found that the [NAD^+^]/[NADH] ratio also increases in cells incubated with low light (Table 4). We found that LH4 synthesis increases even under high light if the cells are grown semiaerobically or under nitrogen-fixing conditions, both conditions that consume reducing power. We also found that LH4 synthesis is somewhat reduced when cells are grown on succinate, which involves reducing the quinone pool in order to be metabolized. These data indicate that LH4 synthesis is regulated by a redox signal that is affected by multiple environmental factors, including light, oxygen, and nutrient availability. This is not unique to R. palustris. LH absorption spectra are also altered in Rhodopseudomonas acidophila grown at identical light intensities but on different carbon sources, which suggests that the LH composition may not only be important under light-limiting conditions (38, 39).

While the exact nature of the redox signal that controls LH4 synthesis is unclear, we found a positive correlation between LH4 synthesis and a more oxidized NAD^+^/NADH pool (Table 4), suggesting that this signal is linked to the redox state of the quinone pool and the amount of available reducing equivalents. LhfE is not predicted to localize to the membrane and does not contain a quinone binding site, so direct interaction between LhfE and the quinone pool seems unlikely but cannot be ruled out. It seems more likely that the redox signal is transmitted to LhfE by thioredoxins or glutaredoxins, key players in the thiol/disulfide redox network. The role of such proteins in regulating LH4 synthesis will be the focus of future studies.

## MATERIALS AND METHODS

### Reagents, bacteria, and culture methods.

Unless otherwise indicated, experiments were conducted with R. palustris wild‐type strain CGA009 or its mutant derivatives. Cells were grown aerobically at 30°C during genetic manipulation on defined mineral medium (PM) (40) agar supplemented with 10 mM succinate. All R. palustris strains were grown anaerobically in PM liquid culture or on plates supplemented with 20 mM acetate or in nitrogen‐fixing medium (NFM) liquid culture or on plates supplemented with 20 mM acetate. NFM is the same as PM but with ammonium sulfate omitted. Liquid growth medium was deaerated and then dispensed into culture tubes in an anaerobic glove box, and the tubes were sealed with rubber stoppers. N_2_ gas was provided in the headspace of sealed culture tubes. Plates were incubated in a GasPak EZ anaerobe container system with indicator (Becton Dickinson). All cultures were initially grown anaerobically with 30 μmol photons/m^2^/s from a 60-W incandescent light bulb (General Electric) and then diluted at least twice into fresh PM medium supplemented with 20 mM acetate and incubated at 30°C with high light intensity or low light intensity (4 μmol photons/m^2^/s) provided from a 15-W incandescent light bulb (General Electric). When indicated, medium was supplemented with 10 mM succinate instead of 20 mM acetate. Semiaerobic cultures were grown as described previously (41) in PM with 20 mM acetate. Escherichia coli S17‐1 was grown in LB medium at 37°C. When appropriate, R. palustris was grown with gentamicin at 100 μg/ml. E. coli cultures were supplemented with gentamicin (20 μg/ml), kanamycin (50 μg/ml), or chloramphenicol (25 μg/ml).

### Genetic manipulation of R. palustris.

All strains and plasmids used are listed in Table S4 in the supplemental material. In-frame deletions of *lhfA*, *lhfD*, and *lhfE* were created by PCR using Phusion High-Fidelity DNA polymerase (New England Biolabs) to amplify a 1-kb fragment upstream of the coding region and 1 kb downstream of the stop codon from purified R. palustris CGA009 genomic DNA. These fragments were introduced into PstI-digested pJQ200SK suicide vector using In-Fusion PCR cloning system (Clontech). Complementing vectors p-*lhfA* or p-*lhfE* were constructed by amplification with Phusion high-fidelity DNA polymerase (New England Biolabs) from purified R. palustris CGA009 genomic DNA. These fragments were introduced into either XbaI- and BamHI-digested pBBRMCS-5 (*lhfA*) or EcoRI-digested pBBPgdh (*lhfE*) using In-Fusion PCR cloning system (Clontech).

10.1128/mBio.02838-19.5 TABLE S4 Strains and plasmids used in this study. Download Table S4, DOC file, 0.09 MB. Copyright © 2019 Fixen et al. 2019 Fixen et al. This content is distributed under the terms of the Creative Commons Attribution 4.0 International license.

The vectors used for allelic exchange of wild-type (WT) *bphP2* for *bphP2*^H532A^ or WT *bphP3* for *bphP3*^H547S^ were constructed by PCR amplification of *bphP2* or *bphP3* using Phusion high-fidelity DNA polymerase (New England Biolabs). The resulting 2.3-kb fragment was incorporated into PstI-digested pJQ200SK using the In-Fusion PCR cloning system (Clontech). Site-directed mutagenesis of the resulting plasmid using the PCR-based QuikChange method (Agilent Technologies) was conducted to introduce the H532A substitution into the *Rp*BphP2 coding sequence or the H547A substitution into the *Rp*BphP3 coding sequence. A similar approach was used to introduce the D70A substitution into the LhfA coding sequence and the C141S, C262S, C318S, C325S, and H185A substitutions into the LhfE coding sequence.

All plasmids were mobilized into R. palustris by conjugation with E. coli S17-1, and double-crossover events for deletions or allelic exchange were achieved using a selection and screening strategy described previously (42). All deletions were verified by PCR, and allelic exchange was verified using PCR and sequencing the resulting PCR product.

### Orthologous gene expression analysis.

Gene-to-gene associations were determined by categorizing genes from different strains into orthologous groups using OrthoMCL (17). First, proteins shared among the 17 strains were searched against the KEGG database, using an E-value cutoff of 1e−05. Next, putative orthologous relationships were identified from reciprocal best hits and a Markov cluster algorithm was applied to create groups of orthologous genes. The expression level of each gene in each orthologous group was determined using Xpression (43), and raw sequencing reads are available in the NCBI Gene Expression Omnibus under accession number GSE59544 (8). DESeq (44) was used to identify orthologous groups that had expression levels of 10 or more and had a statistically significant change in gene expression (i.e., *P* value) less than or equal to 0.05.

### Spectrophotometric analysis.

All spectroscopy was carried out using a Beckman Coulter DU 800 spectrophotometer. Whole-cell absorption spectra of R. palustris grown to an optical density at 660 nm (OD_660_) of ∼0.8 were measured and normalized as described previously (15).

### β-Galactosidase assay.

Construction of a *pucBAd* promoter-*lacZ* fusion was constructed by PCR amplification of the 419-bp intergenic region between *lhfA* and *pucBd* and ligation of this product upstream of the promoterless *lacZ* gene in pHRP309 to create pHRP309-P*_pucBAd_*::*lacZ*. β-Galactosidase activity was measured by a variation of the Miller method as described in reference 45. The rate of increase in the absorbance of 420-nm light due to *o*-nitrophenol formation was measured spectrophotometrically using a Beckman Coulter DU 800 spectrophotometer. Activity was calculated with a millimolar extinction coefficient of 4.5 for *o*-nitrophenol at 420 nm. All activity was normalized to total protein concentrations as determined by the Bio-Rad protein assay kit.

### Protein purification.

pET::*lhfE* was created by PCR amplification of the *lhfE* coding sequence. The resulting product was ligated into NdeI- and BamHI-digested pET28a. Site-directed mutagenesis of pET::*lhfE* was conducted using the PCR-based QuikChange method (Agilent Technologies) to create constructs expressing the LhfE variants, LhfE^C262S^ and LhfE^C318S^. Plasmids encoding the wild-type LhfE or its variants were transformed and expressed in E. coli Rosetta 2(DE3)/pLysS (Novagen). Cells were grown to an OD_600_ of 0.4 to 0.5, and protein expression was induced by the addition of 1 mM isopropyl-β-d-thiogalactopyranoside (IPTG). Cells were then incubated overnight at 16°C with shaking. Cells were harvested by centrifugation at 4,000 rpm for 15 min at 4°C, resuspended in buffer A (20 mM Tris [pH 7.9], 20 mM NaCl), and lysed by sonication. Lysed samples were centrifuged at 15,000 rpm for 30 min at 4°C, and the supernatant was incubated with HisPur cobalt resin (Thermo Fischer Scientific) for 1 h. The resin was washed with increasing amounts of imidazole (20, 50, and 100 mM), and the protein was eluted with 200 and 500 mM imidazole. Purified protein was ultrafiltrated using an Amicon Ultra-15 centrifugal unit with a 10-kDa cutoff and buffer A. Similar to conditions described in reference 28, reducing conditions were created by incubating sample with 1 mM dithiothreitol (DTT) for 30 min at room temperature, and oxidizing conditions were created by incubating the sample with 1 mM K_3_Fe(CN)_6_ for 30 min at room temperature. The protein was analyzed by nonreducing SDS-PAGE and stained with Gelcode Blue stain (Thermo Fisher Scientific).

### Extraction and measurement of NAD^+^ and NADH.

Extraction and quantification of NAD^+^ and NADH were conducted as described in reference 46. Absorbance at 570 nm was measured over time in a 96-well plate in a Tecan Genios pro plate reader.
