# Supplementary figures and images for: Redox Regulation of a Light-Harvesting Antenna Complex in an Anoxygenic Phototroph
Source: mBio. 2019 Nov 26;10(6):e02838-19. doi: 10.1128/mBio.02838-19 (PMC6879726; doi:10.1128/mBio.02838-19)

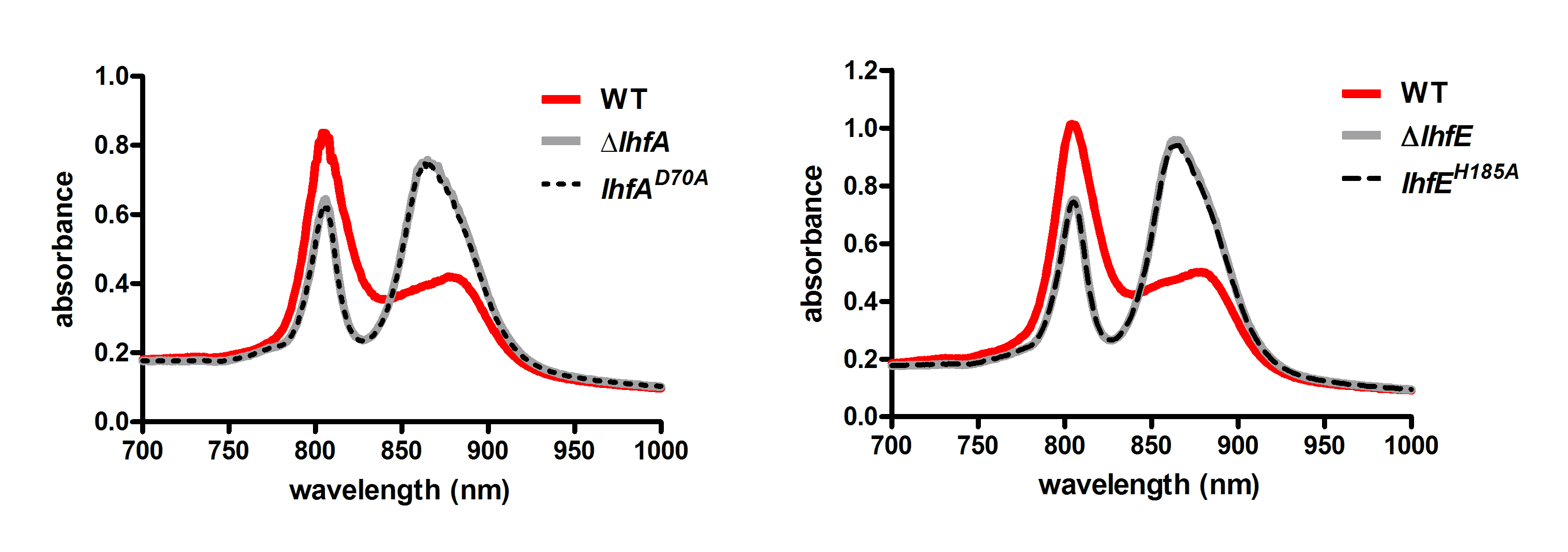

Supplement: FIG S1 [file mBio.02838-19-sf001.tif]
