# Supplementary material for: Redox Regulation of a Light-Harvesting Antenna Complex in an Anoxygenic Phototroph
Source: mBio. 2019 Nov 26;10(6):e02838-19. doi: 10.1128/mBio.02838-19 (PMC6879726; doi:10.1128/mBio.02838-19)
Supplement: TABLE S4 [file mBio.02838-19-st004.doc]

**Table S4. Strains and plasmids** used.

| Strain or plasmid | Genotype, phenotype | Reference,  origin, or description | | | | |
| --- | --- | --- | --- | --- | --- | --- |
| ***R. palustris* strains** |  | | |  |  | |
| CGA009 | Wild-type strain; spontaneous Cm^R^ derivative of CGA001 | | (1) | | |  |
| Δ*bphP2* | CGA009 with an in-frame deletion of *bphP2* (*rpa3015)* | | (2) | | |  |
| Δ*bphP3* | CGA009 with an in-frame deletion of *bphP3* (*rpa3016)* | | (2) | | |  |
| Δ*pucBAd* | CGA009 with an in-frame deletion of *pucBAd* (*rpa3012-rpa3013)* | | (3) | | |  |
| Δ*lhfA* | CGA009 with an in-frame deletion of *lhfA* (*rpa3014*) | | this study | | |  |
| Δ*lhfD* | CGA009 with an in-frame deletion of *lhfD* (*rpa3017*) | | this study | | |  |
| Δ*lhfE* | CGA009 with an in-frame deletion of *lhfE* (*rpa3018*) | | this study | | |  |
| *bphP2*^H532A^ | CGA009 in which the *bphP2^H532A^* mutation was introduced at its native locus using allelic exchange | | this study | | |  |
| *bphP2*^H532A^ Δ*lhfE* | Δ*lhfE* in which the *bphP2^H532A^* mutation was introduced at its native locus using allelic exchange | | this study | | |  |
| *bphP3*^H547A^ | CGA009 in which the *bphP3^H547A^* mutation was introduced at its native locus using allelic exchange | | this study | | |  |
| *bphP2*^H532A^ *bphP3*^H547A^ | CGA009 in which the *bphP2^H532A^* and the *bphP3^H547A^* mutation was introduced at its native locus using allelic exchange | | this study | | |  |
| *lhfA^D70A^* | CGA009 in which the *lhfA^D70A^* mutation was introduced at its native locus using allelic exchange | | this study | | |  |
| *lhfE^H185A^* | CGA009 in which the *lhfE^H185A^* mutation was introduced at its native locus using allelic exchange | | this study | | |  |
| *lhfE^C141S^* | CGA009 in which the *lhfE^C141S^* mutation was introduced at its native locus using allelic exchange | | this study | | |  |
| *lhfE^C262S^* | CGA009 in which the *lhfE^C262S^* mutation was introduced at its native locus using allelic exchange | | this study | | |  |
| *lhfE^C318S^* | CGA009 in which the *lhfE^C318S^* mutation was introduced at its native locus using allelic exchange | | this study | | |  |
| *lhfE^C325S^* | CGA009 in which the *lhfE^C325S^* mutation was introduced at its native locus using allelic exchange | | this study | | |  |
| ***E. coli* strains** |  | |  | | |  |
| DH5α | F^−^ λ^−^ *recA1* Δ(*lacZYA-argF*)*U169 hsdR17 thi-1 gyrA96 supE44 endA1 relA1* Φ80*lacZ*ΔM15 | | Gibco-BRL | | |  |
| S17-1 | *thi pro hdsR hdsM^+^ recA*; chromosomal insertion of RP4-2 (Tc::Mu Km::Tn7) | | (4) | | |  |
| Rosetta 2(DE3)  pLysS | F^-^  *ompT* *hsdS*_B_(r_B_^−^ m_B_^−^) *gal* *dcm* (DE3) pLysSRARE2 (Cam^R^) | | EMB Bioscience | | |  |
| **Plasmids** |  | |  | | |  |
| pJQ200SK | Gm^R^, *sacB*; mobilizable suicide vector | | (5) | | |  |
| pBBRMCS-5 | Gm^R^; mobilizable broad-host-range cloning vector | | (6) | | |  |
| pBBPgdh | Gm^R^; pBBR1MCS-5 with RPA0944 promoter between KpnI and XhoI sites | | (7) | | |  |
| pET28a | Kan^R^; bacterial expression vector with T7lac promoter and N-terminal or C-terminal his-tag | | EMD Biosciences | | |  |
| pHRP309 | IncQ; *lacZ* transcriptional fusion vector | | (8) | | |  |
| pET-*lhfE* | Kan^R^; *lhfE* coding sequence cloned into NdeI/BamHI site of pET28a | | this study | | |  |
| pET-*lhfE*^C262S^ | Kan^R^; pET-*lhfE* encoding C262S amino acid substitution | | this study | | |  |
| pET-*lhfE*^C318S^ | Kan^R^; pET-*lhfE* encoding C262S amino acid substitution | | this study | | |  |
| pJQ-Δ*lhfA* | Gm^R^; in-frame Δ*lhfA* (*rpa3014*) cloned into PstI site of pJQ200SK | | this study | | |  |
| pJQ-Δ*lhfD* | Gm^R^; in-frame Δ*lhfD* (*rpa3017*) cloned into PstI site of pJQ200SK | | this study | | |  |
| pJQ-Δ*lhfE* | Gm^R^; in-frame Δ*lhfE* (*rpa3018*) cloned into PstI site of pJQ200SK | | this study | | |  |
| p-*lhfA* | Gm^R^; *lhfA* cloned into plasmid pBBR1MCS-5 using XbaI and BamHI site | | this study | | |  |
| p-*lhfE* | Gm^R^; *lhfE* with 5’ rbs cloned into the EcoRI site of pBBPgdh | | this study | | |  |
| pJQ-*bphP2*^H532A^ | Gm^R^; *bphP2* encoding H532A amino acid substitution cloned into PstI site of pJQ200SK | | this study | | |  |
| pJQ-*bphP3*^H547A^ | Gm^R^; *bphP3* encoding H547A amino acid substitution cloned into PstI site of pJQ200SK | | this study | | |  |
| pJQ-*lhfA*^D70A^ | Gm^R^; *lhfA* encoding D70A amino acid substitution cloned into PstI site of pJQ200SK | | this study | | |  |
| pJQ-*lhfE*^C141S^ | Gm^R^; *lhfE* encoding C141S amino acid substitution cloned into PstI site of pJQ200SK | | this study | | |  |
| pJQ-*lhfE*^C262S^ | Gm^R^; *lhfE* encoding C262S amino acid substitution cloned into PstI site of pJQ200SK | | this study | | |  |
| pJQ-*lhfE*^C318S^ | Gm^R^; *lhfE* encoding C318S amino acid substitution cloned into PstI site of pJQ200SK | | this study | | |  |
| pJQ-*lhfE*^C325S^ | Gm^R^; *lhfE* encoding C325S amino acid substitution cloned into PstI site of pJQ200SK | | this study | | |  |
| pHRP309-P*_pucBAd_*::*lacZ* | Gm^R^; 419 bp intergenic region between *lhfA* and *pucBd* cloned into XbaI and KpnI site of pHRP309 | | this study | | |  |

**SI References**

1. **Kim M-K**, **Harwood CS**. 1991. Regulation of benzoate-CoA ligase in *Rhodopseudomonas palustris*. FEMS Microbiol Lett **83**:199–203.

2. **Fixen KR**, **Baker AW**, **Stojkovic EA**, **Beatty JT**, **Harwood CS**. 2014. Apo-bacteriophytochromes modulate bacterial photosynthesis in response to low light. Proc Natl Acad Sci USA **111**:E237–44.

3. **Fixen KR**, **Oda Y**, **Harwood CS**. 2016. Clades of photosynthetic bacteria belonging to the genus *Rhodopseudomonas* show marked diversity in light-harvesting antenna complex gene composition and expression. mSystems **1**:e00006–15.

4. **Simon R**, **Priefer U**, **Pühler A**. 1983. A broad host range mobilization system for *in vivo* genetic engineering: transposon mutagenesis in gram-negative bacteria. Nat Biotechnol **1**:784–791.

5. **Quandt J**, **Hynes MF**. 1993. Versatile suicide vectors which allow direct selection for gene replacement in gram-negative bacteria. Gene **127**:15–21.

6. **Kovach ME**, **Phillips RW**, **Elzer PH**, **Roop RM**, **Peterson KM**. 1994. pBBR1MCS: a broad-host-range cloning vector. Biotechniques **16**:800–802.

7. **McKinlay JB**, **Harwood CS**. 2010. Carbon dioxide fixation as a central redox cofactor recycling mechanism in bacteria. Proc Natl Acad Sci USA **107**:11669–11675.

8. **Parales RE**, **Harwood CS**. 1993. Construction and use of a new broad-host-range *lacZ* transcriptional fusion vector, pHRP309, for gram-negative bacteria. Gene **133**:23–30.
